# Supplementary material for: Teacher-rated aggression and co-occurring behaviors and emotional problems among schoolchildren in four population-based European cohorts
Source: PLoS One. 2021 Apr 29;16(4):e0238667. doi: 10.1371/journal.pone.0238667 (PMC8084195; doi:10.1371/journal.pone.0238667)
Supplement: S1 Text — (DOCX) [file pone.0238667.s001.docx]

**S1 Text**

**Finland (FT12)**

*Mean 2015 PISA scores on Science, Reading, Math*: 531, 526, 511

*2015 PISA-reported percentage of youths experiencing ‘frequent’ exposure to bullying:* 9.5% (8.9% OECD average)

*Starting school age*: 7 years old

*School funding*: Schools are all publicly funded (even private schools [comprising 2% of all Finnish pupils] receive public subsidies)

*Special education*: Since the 1970s, there has been no streaming (for children with special needs or ‘gifted’ students), the focus for children with special needs is on providing support as early as possible to prevent the emergence and growth of problems. Since 2010, support has fallen into three categories: general, intensified and special support. Everyone is entitled to general support (part of the everyday teaching and learning process). Intensified and special supports are based on careful assessment and long-span planning in multi-professional teams and on individual education plans for learners.

*Teacher ratings collection*: Parents of the twins (at age 12) were asked to consent to contacting the classroom teacher of each twin to send them the MPNI questionnaire; 87% of parents consented. The MPNI Teacher Rating Form was sent to 2677 class teachers, and 93% responded. Co-twins in 87% of these twin pairs were in the same classrooms and, therefore, rated by the same teacher. Teachers were asked to rate the twin in question, as well as an age-matched peer in the same class. At age 14, the same MPNI Teacher Rating Form as at age 12 was used. Because of the subject-teaching system in the lower secondary schools, each twin (n = 5357) was asked to select one of his/her teachers to complete the ratings; 4726 (88%) twins provided this information. The MPNI Teacher Rating Forms were sent to the teachers (3201) and 3013 (94%) of them completed and returned the forms.

*Twin treatment in schools*: Twins are generally separated into different classrooms. Additionally, in Finland, in the first 5–6 years of schooling it is likely that a child has the same teacher every year, who thus knows them well (for the FT12 age 12 assessment, for example).

**The Netherlands (GENR and NTR)**

*Mean 2015 PISA scores on Science, Reading, Math*: 509, 503, 512

*2015 PISA-reported percentage of youths experiencing ‘frequent’ exposure to bullying:* 3.3% (8.9% OECD average)

*Starting school age*: 4 years old

*School funding*: Schools are publicly funded (even private schools receive public subsidies)

*Special education*: From 1998, special education was available for 4 different categories of pupils: 1) visual impairments, 2) hearing and communicative impairments, 3) physical, intellectual and multiple impairments, and chronically ill pupils and 4) behavioral disorders, severe disabilities, chronically ill (psychiatric) pupils and pupils in pedogogical institutes. Since 2014, the Education That Fits Act provided for more inclusive education of children with special education needs.

*Teacher ratings collection* (GENR): Questionnaires were sent to teachers after asking parents which school their children went to. Parents provided informed consent.

*Teacher ratings collection* (NTR): Parents of twins were asked for permission to approach the teachers of their children. If they consented, they were asked for the names of the school and the teachers were invited next.

*Twin treatment in schools* (NTR): Twins are separated in different classrooms for roughly 50% of pairs.

**The United Kingdom (TEDS)**

*Mean 2015 PISA scores on Science, Reading, Math*: 509, 498, 492

*2015 PISA-reported percentage of youths experiencing ‘frequent’ exposure to bullying:* 14.2% (8.9% OECD average)

*Starting school age*: 4–5 years old

*School funding*: Approximately 90% of pupils are in state-funded primary or secondary schools in England (the rest are in independent and special schools)

*Special education*: In 2014, the Children and Families Act required local authorities to identify and assess the needs of children with special educational needs. Where necessary, they draw up an Education, Health and Care (EHC) plan. Children with an EHC can choose between mainstream or special schools, children without an EHC are in mainstream schools. From 2007–2018, between 14 and 21% of children in the UK had special education needs.

*Teacher ratings collection*: Parents were asked to supply the name and contact details of the main classroom teacher for each twin, at each age. At ages 7 and 9, and for some twins at age 12, the children were in primary school where it is customary for a single teacher to deliver most or all of the curriculum. For twins at secondary school at the time of the age 12 assessment, we asked parents to name the “form teacher” for each twin, this being the teacher having at least daily contact at classroom registration time.

*Twin treatment in schools*: At younger ages (ages 7 and 9 here), twins would likely have had the same teacher.

**References**

European Agency for Special Needs and Inclusive Education. <https://www.european-agency.org/country-information> (Last Accessed: 10 April 2019 for Finland, the Netherlands and the UK)

Eurydice Network on National Education Systems, Education and Youth Policy Analysis Unit in the Education, Audiovisual and Culture Executive Agency (EACEA), European Commission. <https://eacea.ec.europa.eu/national-policies/eurydice/national-description_en> (Last Accessed: 10 April 2019)

OECD (2016), PISA 2015 Results (Volume I): Excellence and Equity in Education, PISA, OECD Publishing, Paris, <https://doi.org/10.1787/9789264266490-en>

OECD (2017), PISA 2015 Results (Volume III): Students' Well-Being, PISA, OECD Publishing, Paris. doi: 10.1787/9789264273856-en

Special Educational Needs in England 2018. Data Insight and Statistics Division, Data Group, Department for Education. <https://assets.publishing.service.gov.uk/government/uploads/system/uploads/attachment_data/file/729208/SEN_2018_Text.pdf>

Schools, pupils and their characteristics: January 2018. Dr. Anastasia Ioannou, Data Insight and Statistics Division, Department for Education. <https://assets.publishing.service.gov.uk/government/uploads/system/uploads/attachment_data/file/719226/Schools_Pupils_and_their_Characteristics_2018_Main_Text.pdf>
