# Supplementary material for: Teacher-rated aggression and co-occurring behaviors and emotional problems among schoolchildren in four population-based European cohorts
Source: PLoS One. 2021 Apr 29;16(4):e0238667. doi: 10.1371/journal.pone.0238667 (PMC8084195; doi:10.1371/journal.pone.0238667)
Supplement: S1 Table — Mean, standard deviation (SD), standard error (SE) and effect sizea summaries by behavioral questionnaire, age, and gender; A. MPNI questionnaire (FT12)b, B. TRF questionnaire (GENR and NTR)b, C. SDQ questionnaire (TEDS)b. (DOCX) [file pone.0238667.s002.docx]

**S1A-S1C Table.** Mean, standard deviation (SD), standard error (SE) and effect size^a^ summaries by behavioral questionnaire, age, and gender

A. MPNI questionnaire (FT12)^b^

| **Age 12** | *Boys (N=1105)* | | *Girls (N=1081)* | |  |
| --- | --- | --- | --- | --- | --- |
|  | Mean (SD) | SE | Mean (SD) | SE | Cohen’s *d*^a^ |
| Aggression | 0.76 (0.68) | 0.02 | 0.50 (0.57) | 0.02 | 0.42 |
| Hyperactivity-Impulsivity | 0.94 (0.79) | 0.02 | 0.46 (0.56) | 0.02 | 0.69 |
| Inattention | 0.94 (0.73) | 0.02 | 0.51 (0.57) | 0.02 | 0.66 |
| Depression | 0.64 (0.50) | 0.01 | 0.71 (0.53) | 0.02 | -0.12 |
| Social Anxiety | 0.78 (0.69) | 0.02 | 0.96 (0.77) | 0.02 | -0.23 |
| Prosocial | 1.67 (0.52) | 0.02 | 1.86 (0.49) | 0.01 | -0.37 |
|  |  |  |  |  |  |
| **Age 14** | *Boys (N=697)* | | *Girls (N=744)* | |  |
|  | Mean (SD) | SE | Mean (SD) | SE | Cohen’s *d*^a^ |
| Aggression | 0.42 (0.56) | 0.02 | 0.24 (0.40) | 0.01 | 0.37 |
| Hyperactivity-Impulsivity | 0.68 (0.75) | 0.03 | 0.34 (0.54) | 0.02 | 0.52 |
| Inattention | 0.84 (0.72) | 0.03 | 0.45 (0.57) | 0.02 | 0.61 |
| Depression^b^ | 0.53 (0.49) | 0.02 | 0.57 (0.49) | 0.02 | -0.09 |
| Social Anxiety | 0.71 (0.72) | 0.03 | 0.88 (0.80) | 0.03 | -0.21 |
| Prosocial | 1.69 (0.51) | 0.02 | 1.91 (0.47) | 0.02 | -0.45 |

^a^Positive values indicate greater means in boys compared to girls, and negative values greater means for girls

^b^All mean gender differences (examined using T-tests) are significant (p<0.01), except for that indicated

B. TRF questionnaire (GENR and NTR)^b^

| **GENR** |  |  | |  |  |  |
| --- | --- | --- | --- | --- | --- | --- |
| **Age 7** | *Boys (N=2270)* | | | *Girls (N=2242)* | |  |
|  | Mean (SD) | | SE | Mean (SD) | SE | Cohen’s *d*^a^ |
| Aggressive Behavior | 3.60 (6.18) | | 0.13 | 1.48 (3.44) | 0.07 | 0.42 |
| Attention Problems | 9.09 (9.95) | | 0.21 | 4.34 (6.59) | 0.14 | 0.56 |
| Rule-Breaking Behavior | 1.18 (2.16) | | 0.05 | 0.52 (1.31) | 0.03 | 0.37 |
| Anxious/Depressed | 1.84 (2.84) | | 0.06 | 1.57 (2.60) | 0.05 | 0.10 |
| Somatic Problems | 0.36 (1.04) | | 0.02 | 0.40 (1.03) | 0.02 | -0.04 |
| Withdrawn/Depressed | 1.16 (1.88) | | 0.04 | 0.95 (1.71) | 0.04 | 0.12 |
| Social Problems | 1.46 (2.28) | | 0.05 | 0.94 (1.73) | 0.04 | 0.26 |
| Thought Problems | 0.72 (1.71) | | 0.04 | 0.29 (0.95) | 0.02 | 0.31 |
|  |  | |  |  |  |  |
| **NTR** |  | |  |  |  |  |
| **Age 7** | *Boys (N=3416)* | | | *Girls (N=3518)* | |  |
|  | Mean (SD) | | SE | Mean (SD) | SE | Cohen’s *d*^a^ |
| Aggressive Behavior | 3.24 (5.25) | | 0.09 | 1.51 (3.06) | 0.05 | 0.40 |
| Attention Problems | 8.62 (8.76) | | 0.15 | 4.51 (6.01) | 0.10 | 0.55 |
| Rule-Breaking Behavior | 0.75 (1.52) | | 0.03 | 0.36 (0.98) | 0.02 | 0.31 |
| Anxious/Depressed | 2.58 (3.13) | | 0.05 | 2.43 (2.89) | 0.05 | 0.05 |
| Somatic Problems | 0.30 (0.83) | | 0.01 | 0.37 (0.94) | 0.02 | -0.09 |
| Withdrawn/Depressed | 1.35 (1.98) | | 0.03 | 1.20 (1.81) | 0.03 | 0.08 |
| Social Problems | 1.65 (2.28) | | 0.04 | 1.17 (1.84) | 0.03 | 0.23 |
| Thought Problems | 0.62 (1.35) | | 0.02 | 0.30 (0.84) | 0.01 | 0.28 |
|  |  | |  |  |  |  |
| **Age 10** | *Boys (N=3264)* | | | *Girls (N=3318)* | |  |
|  | Mean (SD) | | SE | Mean (SD) | SE | Cohen’s *d*^a^ |
| Aggressive Behavior | 3.54 (5.62) | | 0.10 | 1.67 (3.43) | 0.06 | 0.40 |
| Attention Problems | 9.05 (8.77) | | 0.15 | 4.58 (6.01) | 0.10 | 0.59 |
| Rule-Breaking Behavior | 0.91 (1.72) | | 0.03 | 0.39 (1.03) | 0.02 | 0.37 |
| Anxious/Depressed^b^ | 2.66 (3.15) | | 0.06 | 2.60 (3.15) | 0.05 | 0.02 |
| Somatic Problems | 0.34 (0.97) | | 0.02 | 0.39 (1.02) | 0.02 | -0.05 |
| Withdrawn/Depressed | 1.54 (2.18) | | 0.04 | 1.33 (1.94) | 0.03 | 0.10 |
| Social Problems | 1.70 (2.48) | | 0.04 | 1.22 (2.05) | 0.04 | 0.22 |
| Thought Problems | 0.64 (1.36) | | 0.02 | 0.32 (0.90) | 0.02 | 0.28 |
|  |  | |  |  |  |  |
| **Age 12** | *Boys (N=2477)* | | | *Girls (N=2576)* | |  |
|  | Mean (SD) | | SE | Mean (SD) | SE | Cohen’s *d*^a^ |
| Aggressive Behavior | 3.26 (5.48) | | 0.11 | 1.38 (2.95) | 0.06 | 0.43 |
| Attention Problems | 8.07 (8.56) | | 0.17 | 3.72 (5.27) | 0.10 | 0.61 |
| Rule-Breaking Behavior | 0.90 (1.81) | | 0.04 | 0.35 (0.97) | 0.02 | 0.38 |
| Anxious/Depressed^b^ | 2.33 (3.04) | | 0.06 | 2.38 (3.06) | 0.06 | -0.01 |
| Somatic Problems^b^ | 0.31 (0.93) | | 0.02 | 0.30 (0.90) | 0.02 | 0.01 |
| Withdrawn/Depressed | 1.44 (2.10) | | 0.04 | 1.31 (2.00) | 0.04 | 0.06 |
| Social Problems | 1.49 (2.34) | | 0.05 | 1.08 (2.00) | 0.04 | 0.19 |
| Thought Problems | 0.48 (1.21) | | 0.02 | 0.27 (0.88) | 0.02 | 0.20 |

^a^Positive values indicate greater means in boys compared to girls, and negative values greater means for girls

^b^All mean gender differences (examined using T-tests) are significant (p<0.05), except for those indicated

C. SDQ questionnaire (TEDS)^b^

| **Age 7** | *Boys (N=2834)* | | *Girls (N=2987)* | |  |
| --- | --- | --- | --- | --- | --- |
|  | Mean (SD) | SE | Mean (SD) | SE | Cohen’s *d*^a^ |
| Aggressive Behavior | 0.96 (1.58) | 0.03 | 0.50 (1.11) | 0.02 | 0.34 |
| Hyperactivity | 3.64 (2.90) | 0.05 | 2.26 (2.24) | 0.04 | 0.53 |
| Anxiety^b^ | 1.27 (1.74) | 0.03 | 1.35 (1.82) | 0.03 | -0.05 |
| Peer Problems | 1.12 (1.50) | 0.03 | 0.97 (1.40) | 0.03 | 0.10 |
| Prosocial | 6.67 (2.45) | 0.05 | 7.83 (2.12) | 0.04 | -0.51 |
|  |  |  |  |  |  |
| **Age 9** | *Boys (N=1295)* | | *Girls (N=1467)* | |  |
|  | Mean (SD) | SE | Mean (SD) | SE | Cohen’s *d*^a^ |
| Aggressive Behavior | 0.77 (1.49) | 0.04 | 0.34 (0.90) | 0.02 | 0.35 |
| Hyperactivity | 3.29 (2.69) | 0.07 | 1.96 (2.02) | 0.05 | 0.56 |
| Anxiety | 1.28 (1.80) | 0.05 | 1.43 (1.87) | 0.05 | -0.08 |
| Peer Problems | 0.95 (1.52) | 0.04 | 0.76 (1.30) | 0.03 | 0.14 |
| Prosocial | 6.78 (2.40) | 0.07 | 8.15 (1.96) | 0.05 | -0.63 |
|  |  |  |  |  |  |
| **Age 12** | *Boys (N=2168)* | | *Girls (N=2477)* | |  |
|  | Mean (SD) | SE | Mean (SD) | SE | Cohen’s *d*^a^ |
| Aggressive Behavior | 0.81 (1.46) | 0.03 | 0.39 (0.99) | 0.02 | 0.34 |
| Hyperactivity | 2.93 (2.75) | 0.06 | 1.52 (1.92) | 0.04 | 0.60 |
| Anxiety^b^ | 1.26 (1.84) | 0.04 | 1.20 (1.75) | 0.04 | 0.03 |
| Peer Problems | 1.24 (1.75) | 0.04 | 0.86 (1.42) | 0.03 | 0.24 |
| Prosocial | 7.40 (2.17) | 0.05 | 8.50 (1.82) | 0.04 | -0.55 |

^a^Positive values indicate greater means in boys compared to girls, and negative values greater means for girls

^b^All mean gender differences (examined using T-tests) are significant (p<0.05), except for those indicated
