# Supplementary material for: Teacher-rated aggression and co-occurring behaviors and emotional problems among schoolchildren in four population-based European cohorts
Source: PLoS One. 2021 Apr 29;16(4):e0238667. doi: 10.1371/journal.pone.0238667 (PMC8084195; doi:10.1371/journal.pone.0238667)
Supplement: S2 Table — Pearson correlations of all behavioral subscales with each other by behavioral questionnaire, age, and gender; A. MPNI questionnaire (FT12)*, B. TRF questionnaire (GENR and NTR)*; C. SDQ questionnaire (TEDS). (DOCX) [file pone.0238667.s003.docx]

**S2A-S2C Table.** Pearson correlations of all behavioral subscales with each other by behavioral questionnaire, age, and gender

A. MPNI questionnaire (FT12)*

| **Age 12** |  |  |  |  |  |  |  |  |  |  |  |
| --- | --- | --- | --- | --- | --- | --- | --- | --- | --- | --- | --- |
| **BOYS** | Hyp-Imp | Inatt | Dep | Soc Anx | Prosoc | **GIRLS** | Hyp-Imp | Inatt | Dep | Soc Anx | Prosoc |
| Aggression | **0.75** | **0.56** | **0.25** | -0.06 | **-0.54** | Aggression | **0.70** | **0.48** | **0.29** | -0.05 | **-0.40** |
| Hyp-Imp |  | **0.70** | **0.17** | **-0.12** | **-0.48** | Hyp-Imp |  | **0.61** | **0.18** | **-0.15** | **-0.30** |
| Inatt |  |  | **0.25** | **0.07** | **-0.58** | Inatt |  |  | **0.26** | 0.06 | **-0.45** |
| Dep |  |  |  | **0.59** | **-0.23** | Dep |  |  |  | **0.53** | **-0.32** |
| Soc Anx |  |  |  |  | **-0.17** | Soc Anx |  |  |  |  | **-0.28** |
|  |  |  |  |  |  |  |  |  |  |  |  |
| **Age 14** |  |  |  |  |  |  |  |  |  |  |  |
| **BOYS** | Hyp-Imp | Inatt | Dep | Soc Anx | Prosoc | **GIRLS** | Hyp-Imp | Inatt | Dep | Soc Anx | Prosoc |
| Aggression | **0.71** | **0.51** | **0.16** | **-0.10** | **-0.44** | Aggression | **0.69** | **0.55** | **0.26** | -0.02 | **-0.33** |
| Hyp-Imp |  | **0.71** | 0.06 | **-0.20** | **-0.44** | Hyp-Imp |  | **0.74** | **0.15** | **-0.16** | **-0.29** |
| Inatt |  |  | **0.16** | 0.01 | **-0.61** | Inatt |  |  | **0.19** | -0.02 | **-0.44** |
| Dep |  |  |  | **0.60** | **-0.21** | Dep |  |  |  | **0.62** | **-0.28** |
| Soc Anx |  |  |  |  | **-0.14** | Soc Anx |  |  |  |  | **-0.28** |

*all bold correlations are significant (p<0.05)

Abbreviations: Dep=depression, Hyp-Imp=hyperactivity/impulsivity, Inatt=inattention, Soc Anx=social anxiety, Prosoc=prosocial

B. TRF questionnaire (GENR and NTR)*

| **GEN-R** |  |  |  |  |  |  |  |  |  |  |  |  |  |  |  |
| --- | --- | --- | --- | --- | --- | --- | --- | --- | --- | --- | --- | --- | --- | --- | --- |
| **Age 7** |  |  |  |  |  |  |  |  |  |  |  |  |  |  |  |
| **BOYS** | AP | RB | ANX | SOM | WIT | SOC | TP | **GIRLS** | AP | RB | ANX | SOM | WIT | SOC | TP |
| AGG | 0.75 | 0.80 | 0.39 | 0.19 | 0.27 | 0.65 | 0.59 | AGG | 0.69 | 0.74 | 0.32 | 0.14 | 0.21 | 0.65 | 0.49 |
| AP |  | 0.67 | 0.37 | 0.23 | 0.34 | 0.66 | 0.60 | AP |  | 0.61 | 0.35 | 0.23 | 0.31 | 0.66 | 0.58 |
| RB |  |  | 0.34 | 0.26 | 0.27 | 0.57 | 0.54 | RB |  |  | 0.22 | 0.15 | 0.21 | 0.49 | 0.43 |
| ANX |  |  |  | 0.41 | 0.52 | 0.55 | 0.52 | ANX |  |  |  | 0.32 | 0.56 | 0.56 | 0.50 |
| SOM |  |  |  |  | 0.31 | 0.32 | 0.32 | SOM |  |  |  |  | 0.25 | 0.26 | 0.26 |
| WIT |  |  |  |  |  | 0.45 | 0.41 | WIT |  |  |  |  |  | 0.45 | 0.37 |
| SOC |  |  |  |  |  |  | 0.61 | SOC |  |  |  |  |  |  | 0.61 |
|  |  |  |  |  |  |  |  |  |  |  |  |  |  |  |  |
| **NTR** |  |  |  |  |  |  |  |  |  |  |  |  |  |  |  |
| **Age 7** |  |  |  |  |  |  |  |  |  |  |  |  |  |  |  |
| **BOYS** | AP | RB | ANX | SOM | WIT | SOC | TP | **GIRLS** | AP | RB | ANX | SOM | WIT | SOC | TP |
| AGG | 0.69 | 0.71 | 0.29 | 0.15 | 0.18 | 0.62 | 0.47 | AGG | 0.62 | 0.63 | 0.26 | 0.17 | 0.15 | 0.60 | 0.39 |
| AP |  | 0.57 | 0.23 | 0.23 | 0.23 | 0.60 | 0.51 | AP |  | 0.46 | 0.24 | 0.26 | 0.23 | 0.57 | 0.46 |
| RB |  |  | 0.19 | 0.12 | 0.18 | 0.48 | 0.37 | RB |  |  | 0.18 | 0.12 | 0.14 | 0.46 | 0.34 |
| ANX |  |  |  | 0.18 | 0.48 | 0.49 | 0.41 | ANX |  |  |  | 0.25 | 0.52 | 0.48 | 0.38 |
| SOM |  |  |  |  | 0.18 | 0.25 | 0.19 | SOM |  |  |  |  | 0.37 | 0.28 | 0.23 |
| WIT |  |  |  |  |  | 0.41 | 0.36 | WIT |  |  |  |  |  | 0.37 | 0.33 |
| SOC |  |  |  |  |  |  | 0.53 | SOC |  |  |  |  |  |  | 0.44 |
|  |  |  |  |  |  |  |  |  |  |  |  |  |  |  |  |
| **Age 10** |  |  |  |  |  |  |  |  |  |  |  |  |  |  |  |
| **BOYS** | AP | RB | ANX | SOM | WIT | SOC | TP | **GIRLS** | AP | RB | ANX | SOM | WIT | SOC | TP |
| AGG | 0.70 | 0.74 | 0.36 | 0.15 | 0.17 | 0.60 | 0.47 | AGG | 0.66 | 0.67 | 0.33 | 0.17 | 0.19 | 0.61 | 0.46 |
| AP |  | 0.58 | 0.33 | 0.2 | 0.24 | 0.58 | 0.53 | AP |  | 0.56 | 0.33 | 0.20 | 0.26 | 0.55 | 0.48 |
| RB |  |  | 0.27 | 0.12 | 0.18 | 0.49 | 0.40 | RB |  |  | 0.23 | 0.16 | 0.18 | 0.45 | 0.38 |
| ANX |  |  |  | 0.29 | 0.53 | 0.55 | 0.47 | ANX |  |  |  | 0.32 | 0.51 | 0.56 | 0.45 |
| SOM |  |  |  |  | 0.24 | 0.26 | 0.24 | SOM |  |  |  |  | 0.18 | 0.25 | 0.23 |
| WIT |  |  |  |  |  | 0.42 | 0.39 | WIT |  |  |  |  |  | 0.45 | 0.31 |
| SOC |  |  |  |  |  |  | 0.52 | SOC |  |  |  |  |  |  | 0.50 |
|  |  |  |  |  |  |  |  |  |  |  |  |  |  |  |  |
| **Age 12** |  |  |  |  |  |  |  |  |  |  |  |  |  |  |  |
| **BOYS** | AP | RB | ANX | SOM | WIT | SOC | TP | **GIRLS** | AP | RB | ANX | SOM | WIT | SOC | TP |
| AGG | 0.73 | 0.78 | 0.38 | 0.16 | 0.18 | 0.54 | 0.46 | AGG | 0.67 | 0.67 | 0.31 | 0.18 | 0.18 | 0.53 | 0.38 |
| AP |  | 0.64 | 0.38 | 0.23 | 0.24 | 0.51 | 0.47 | AP |  | 0.55 | 0.33 | 0.22 | 0.27 | 0.49 | 0.44 |
| RB |  |  | 0.30 | 0.15 | 0.19 | 0.43 | 0.42 | ANX |  |  | 0.24 | 0.13 | 0.16 | 0.42 | 0.32 |
| ANX |  |  |  | 0.33 | 0.52 | 0.57 | 0.52 | WIT |  |  |  | 0.34 | 0.53 | 0.53 | 0.47 |
| SOM |  |  |  |  | 0.25 | 0.25 | 0.25 | SOM |  |  |  |  | 0.24 | 0.31 | 0.28 |
| WIT |  |  |  |  |  | 0.51 | 0.36 | SOC |  |  |  |  |  | 0.48 | 0.35 |
| SOC |  |  |  |  |  |  | 0.51 | RB |  |  |  |  |  |  | 0.46 |

*all correlations are significant (p<0.05)

AGG=aggressive behavior, AP=attention problems, ANX=anxious/depressed, WIT=withdrawn/depressed, SOM=somatic complaints, SOC=social problems, RB=rule-breaking behavior, TP=thought problems

C. SDQ questionnaire (TEDS)*

| **Age 7** |  |  |  |  |  |  |  |  |  |
| --- | --- | --- | --- | --- | --- | --- | --- | --- | --- |
| **BOYS** | Hyperactivity | Anxiety | Peer Problems | Prosocial | **GIRLS** | Hyperactivity | Anxiety | Peer Problems | Prosocial |
| Aggressive Behavior | 0.56 | 0.17 | 0.38 | -0.50 | Aggressive Behavior | 0.50 | 0.13 | 0.32 | -0.44 |
| Hyperactivity |  | 0.19 | 0.33 | -0.44 | Hyperactivity |  | 0.21 | 0.32 | -0.38 |
| Anxiety |  |  | 0.32 | -0.10 | Anxiety |  |  | 0.35 | -0.12 |
| Peer Problems |  |  |  | -0.35 | Peer Problems |  |  |  | -0.36 |
|  |  |  |  |  |  |  |  |  |  |
| **Age 9** |  |  |  |  |  |  |  |  |  |
| **BOYS** | Hyperactivity | Anxiety | Peer Problems | Prosocial | **GIRLS** | Hyperactivity | Anxiety | Peer Problems | Prosocial |
| Aggressive Behavior | 0.51 | 0.17 | 0.42 | -0.50 | Aggressive Behavior | 0.47 | 0.10 | 0.38 | -0.43 |
| Hyperactivity |  | 0.23 | 0.30 | -0.43 | Hyperactivity |  | 0.22 | 0.32 | -0.44 |
| Anxiety |  |  | 0.37 | -0.09 | Anxiety |  |  | 0.34 | -0.06 |
| Peer Problems |  |  |  | -0.33 | Peer Problems |  |  |  | -0.35 |
|  |  |  |  |  |  |  |  |  |  |
| **Age 12** |  |  |  |  |  |  |  |  |  |
| **BOYS** | Hyperactivity | Anxiety | Peer Problems | Prosocial | **GIRLS** | Hyperactivity | Anxiety | Peer Problems | Prosocial |
| Aggressive Behavior | 0.58 | 0.19 | 0.27 | -0.48 | Aggressive Behavior | 0.50 | 0.21 | 0.26 | -0.43 |
| Hyperactivity |  | 0.19 | 0.19 | -0.49 | Hyperactivity |  | 0.22 | 0.21 | -0.42 |
| Anxiety |  |  | 0.49 | -0.11 | Anxiety |  |  | 0.45 | -0.17 |
| Peer Problems |  |  |  | -0.30 | Peer Problems |  |  |  | -0.33 |

*all correlations are significant (p<0.05)
