# Supplementary material for: Teacher-rated aggression and co-occurring behaviors and emotional problems among schoolchildren in four population-based European cohorts
Source: PLoS One. 2021 Apr 29;16(4):e0238667. doi: 10.1371/journal.pone.0238667 (PMC8084195; doi:10.1371/journal.pone.0238667)
Supplement: S3 Table — Modeled separately by behavioral questionnaire, age wave of data collection, and gender; A. MPNI questionnaire (FT12), B. TRF questionnaire (GENR and NTR), C. SDQ questionnaire (TEDS). (DOCX) [file pone.0238667.s004.docx]

**S3A-S3C Table.** Linear regression summaries of standardized multiple independent variable models. Modeled separately by behavioral questionnaire, age wave of data collection, and gender.

A. MPNI questionnaire (FT12)

| *Age 12 Boys (N=1105)* | |  |  | *Age 12 Girls (N=1081)* | |  |  | Gender interaction model | |
| --- | --- | --- | --- | --- | --- | --- | --- | --- | --- |
| **Model Variable** | **Agg β** | **95% CI** | **R^2^** | **Model Variable** | **Agg β** | **95% CI** | **R^2^** | **Interaction term, p** | **R^2^** |
| Hyp-Imp | 0.61 | 0.57, 0.65 | 0.61 | Hyp-Imp | 0.71 | 0.67, 0.76 | 0.54 | 0.00 | 0.60 |
| Depression | 0.10 | 0.06, 0.14 |  | Depression | 0.10 | 0.07, 0.13 |  | 0.97 |  |
| Prosocial | -0.22 | -0.27, -0.18 |  | Prosocial | -0.19 | -0.22, -0.16 |  | 0.06 |  |
|  |  |  |  |  |  |  |  |  |  |
| *Age 14 Boys (N=697)* | |  |  | *Age 14 Girls (N=744)* | |  |  | Gender interaction model | |
| **Model Variable** | **Agg β** | **95% CI** | **R^2^** | **Model Variable** | **Agg β** | **95% CI** | **R^2^** | **Interaction term, p** | **R^2^** |
| Hyp-Imp | 0.66 | 0.60, 0.72 | 0.53 | Hyp-Imp | 0.64 | 0.59, 0.70 | 0.51 | 0.74 | 0.54 |
| Depression | 0.12 | 0.05, 0.18 |  | Depression | 0.11 | 0.06, 0.15 |  | 0.80 |  |
| Prosocial | -0.15 | -0.22, -0.09 |  | Prosocial | -0.10 | -0.15, -0.05 |  | 0.19 |  |

Abbreviations: Agg=aggression, CI=confidence interval, Hyp-Imp=hyperactivity–impulsivity

B. TRF questionnaire (GENR and NTR)

| **GEN-R** *Age 7 Boys (N=2270)* | | |  | *Age 7 Girls (N=2242)* | |  |  | Gender interaction model | |
| --- | --- | --- | --- | --- | --- | --- | --- | --- | --- |
| **Model Variable** | **Agg β** | **95% CI** | **R^2^** | **Model Variable** | **Agg β** | **95% CI** | **R^2^** | **Interaction term, p** | **R^2^** |
| Attention Problems | 0.75 | 0.72, 0.78 | 0.57 | Attention Problems | 0.59 | 0.57, 0.62 | 0.49 | <0.001 | 0.57 |
| Anxious/Depressed | 0.15 | 0.11, 0.18 |  | Anxious/Depressed | 0.06 | 0.04, 0.08 |  | <0.001 |  |
|  |  |  |  |  |  |  |  |  |  |
| **NTR** *Age 7 Boys (N=3416)* | | |  | *Age 7 Girls (N=3518)* | |  |  | Gender interaction model | |
| **Model Variable** | **Agg β** | **95% CI** | **R^2^** | **Model Variable** | **Agg β** | **95% CI** | **R^2^** | **Interaction term, p** | **R^2^** |
| Attention Problems | 0.70 | 0.68, 0.73 | 0.50 | Attention Problems | 0.53 | 0.51, 0.56 | 0.40 | <0.001 | 0.49 |
| Anxious/Depressed | 0.16 | 0.13, 0.19 |  | Anxious/Depressed | 0.09 | 0.07, 0.11 |  | <0.001 |  |
|  |  |  |  |  |  |  |  |  |  |
| *Age 10 Boys (N=3264)* | |  |  | *Age 10 Girls (N=3318)* | |  |  | Gender interaction model | |
| **Model Variable** | **Agg β** | **95% CI** | **R^2^** | **Model Variable** | **Agg β** | **95% CI** | **R^2^** | **Interaction term, p** | **R^2^** |
| Attention Problems | 0.69 | 0.66, 0.71 | 0.50 | Attention Problems | 0.58 | 0.55, 0.60 | 0.45 | <0.001 | 0.51 |
| Anxious/Depressed | 0.17 | 0.14, 0.20 |  | Anxious/Depressed | 0.10 | 0.08, 0.12 |  | <0.001 |  |
|  |  |  |  |  |  |  |  |  |  |
| *Age 12 Boys (N=2477)* | |  |  | *Age 12 Girls (N=2576)* | |  |  | Gender interaction model | |
| **Model Variable** | **Agg β** | **95% CI** | **R^2^** | **Model Variable** | **Agg β** | **95% CI** | **R^2^** | **Interaction term, p** | **R^2^** |
| Attention Problems | 0.73 | 0.70, 0.76 | 0.55 | Attention Problems | 0.59 | 0.56, 0.62 | 0.46 | <0.001 | 0.55 |
| Anxious/Depressed | 0.15 | 0.11, 0.18 |  | Anxious/Depressed | 0.07 | 0.05, 0.09 |  | <0.001 |  |

Abbreviations: Agg=aggression, CI=confidence interval

C. SDQ questionnaire (TEDS)

| *Age 7 Boys (N=2834)* | |  |  | *Age 7 Girls (N=2987)* | |  |  | Gender interaction model | |
| --- | --- | --- | --- | --- | --- | --- | --- | --- | --- |
| **Model Variable** | **Agg β** | **95% CI** | **R^2^** | **Model Variable** | **Agg β** | **95% CI** | **R^2^** | **Interaction term, p** | **R^2^** |
| Hyperactivity | 0.43 | 0.40, 0.47 | 0.39 | Hyperactivity | 0.38 | 0.35, 0.41 | 0.32 | 0.027 | 0.39 |
| Anxiety | 0.07 | 0.04, 0.11 |  | Anxiety | 0.01 | -0.01, 0.04 |  | 0.005 |  |
| Prosocial | -0.35 | -0.39, -0.32 |  | Prosocial | -0.26 | -0.29, -0.23 |  | <0.001 |  |
|  |  |  |  |  |  |  |  |  |  |
| *Age 9 Boys (N=1295)* | |  |  | *Age 9 Girls (N=1467)* | |  |  | Gender interaction model | |
| **Model Variable** | **Agg β** | **95% CI** | **R^2^** | **Model Variable** | **Agg β** | **95% CI** | **R^2^** | **Interaction term, p** | **R^2^** |
| Hyperactivity | 0.40 | 0.34, 0.45 | 0.36 | Hyperactivity | 0.31 | 0.26, 0.35 | 0.28 | 0.014 | 0.36 |
| Anxiety | 0.07 | 0.01, 0.12 |  | Anxiety | 0.01 | -0.03, 0.04 |  | 0.060 |  |
| Prosocial | -0.38 | -0.44, -0.33 |  | Prosocial | -0.23 | -0.27, -0.19 |  | <0.001 |  |
|  |  |  |  |  |  |  |  |  |  |
| *Age 12 Boys (N=2168)* | |  |  | *Age 12 Girls (N=2477)* | |  |  | Gender interaction model | |
| **Model Variable** | **Agg β** | **95% CI** | **R^2^** | **Model Variable** | **Agg β** | **95% CI** | **R^2^** | **Interaction term, p** | **R^2^** |
| Hyperactivity | 0.45 | 0.41, 0.49 | 0.40 | Hyperactivity | 0.37 | 0.33, 0.40 | 0.32 | 0.003 | 0.39 |
| Anxiety | 0.09 | 0.06, 0.13 |  | Anxiety | 0.07 | 0.04, 0.09 |  | 0.291 |  |
| Prosocial | -0.28 | -0.32, -0.24 |  | Prosocial | -0.23 | -0.26, -0.20 |  | 0.091 |  |

Abbreviations: Agg=aggression, CI=confidence interval
