# Supplementary material for: Teacher-rated aggression and co-occurring behaviors and emotional problems among schoolchildren in four population-based European cohorts
Source: PLoS One. 2021 Apr 29;16(4):e0238667. doi: 10.1371/journal.pone.0238667 (PMC8084195; doi:10.1371/journal.pone.0238667)
Supplement: Attachment — Submitted filename: PONE-D-20-29433_reviewer_231120_reviewer.docx [file pone.0238667.s005.docx]

Thank you very much for this interesting paper. The authors show results on the epidemiology of teacher rated aggressive behavior in school children based on an impressive data set. This paper uses a large, international sample which gives interesting findings on

mean levels of teacher rated aggressive behavior and its associations with internalizing / externalizing behavior and prosocial behavior in 7- to 14-year-old students. It seems that this valuable data has also been analyzed in a longitudinal manner in other papers.

However, bevor publishing I highly suggest to discuss the following major aspects more deeply:

**Abstract:**

I suggest to explicitly mention the overall aim of this study within the abstract.

I highly recommend to define aggressive behavior within the abstract. As you mention within your introduction there are different types of aggressive behavior. However, the questionnaires you used may assess only parts of the aggression construct.

Please include information on sample age and school types (if applicable).

**Introduction:**

As mentioned above, you correctly describe the more complex construct of aggression. However, I do not see that you actually assessed all subtypes. Please specify what kind of aggression you are referring to. In addition, some subtypes may be assessed less valid and reliable within a teacher rating (e.g. relational/ indirect aggression). I suggest to include literature on what kind of aggressive behavior can be reliably assessed though teacher ratings. Also, consider to mention this in your limitation section.

Page 8, second sentence “Furthermore, children with aggression and comorbidities often have more than one comorbidity and worse prognosis and poorer outcomes …”. Please specify, worse than what? Poorer outcomes with respect to what?

Page 8, third sentence (“…regarding the school setting”). Please underlie your statement with literature. What about literature on bullying within schools. In my understanding most studies rely on population-based samples. There are few studies comparing population-based samples with clinical samples (see FemNAT-CD study as one recent example including clinical samples).

**Materials and methods**

Please specify „handicap or illness that interferes with daily functioning“. How has this been assessed?

Did the excluded sample due to missing values differ to the included sample with respect to any characteristics? E.g., IQ, SES, teacher or child characteristics, internalizing/externalizing behavior?

**Cohort description:**

Do you have any background information on the samples: IQ, SES, single parent household, psychiatric history?

Do the samples differ with respect to IQ, SES, etc.

If I understood correctly you included some children twice in your study for different age groups, as your data stems from longitudinal studies. Do results differ, if you include each child only once? Can you control for this in your model?

It would be highly interesting to use teacher ratings on aggression as a predictor to later aggressive behavior longitudinally.

How did you get teacher information? For how long did the teacher know the child? Do you have any information on the teachers and schools?

**Study questionnaires**

Please consider sharing information on the questionnaires and subscales psychometrics (Conbachs alpha etc.,)

I suggest to give more information on what kind of aggressive behavior has been assessed through those questionnaires. Please give item examples.

In my understanding conduct problems include more behavioristics than just aggression. Please specify.

**Analysis**

Please consider to control for study site. Especially, as you mention later, that gender differences in internalizing subscale differed between studies. Also, I suggest to control for IQ.

Did you include aggression items within the overall externalizing problem scale? If so, do I understand correctly, that you include items on aggression both in your dependent and independent variables?

How exactly did you test for gender interaction? What was your model?

**Results**

Please check with the journal specifications: I suggest to include r = and N =, etc.

Page 15, information on gender differences in internalizing problems compared between studies: I suggest to discuss the different findings. This may be a reason to use site as random effect within the model.

**Discussion**

I suggest to discuss more deeply the following aspects:

- What types of aggression are the results referring to? I suggest to look into the following literature: Ackermann, K., Kirchner, M., Bernhard, A. *et al.* Relational Aggression in Adolescents with Conduct Disorder: Sex Differences and Behavioral Correlates. *J Abnorm Child Psychol* 47, 1625–1637 (2019). https://doi.org/10.1007/s10802-019-00541-6
- Do you think teacher reports on internalizing behavior are reliable? May it be more appropriate to use self-ratings for internalizing behavior? Do you have any self or parent ratings with which you could compare results?
- I agree that teachers are a valuable and most likely the most reliable informant on aggressive behavior. However, can you state the following based on your results? (“However, girls were less likely than boys to be identified by teachers as disruptive and referred for treatment “.) Can you say that, although you did not compare with self- and parent ratings? Maybe girls are just less aggressive? Maybe, because relational aggression is less likely to be observed by teachers/ others?
- In addition, page 22 (“Teachers are a valuable resource for identifying children in need of specific support and possibly in referral or diagnostic aspects”). I agree, but can you say this along this data? How can you make sure that they capture most children in need of psychological help as you did not compare with a clinical sample?
- Could the teacher rating explain the moderate correlation between aggression and internalizing behavior? As internalizing behavior may not be as visible (cognitions rather than behavior) as externalizing behavior?
- You describe aggression dimensional and not on a clinical level. Therefore, are you able to show with your results that higher scores on internalizing behavior may be a „protective factor“ for externalizing behavior? Is there literature to this?
- You mention highly interesting results on prosocial behavior and future outcomes. Consider to discuss literature on CU traits to this highly interesting finding.
- Page 21. Please explain the following statement: “Overall, teacher-based correlations of aggressive behavior with co-occurring behaviors are higher for externalizing problems and lower for internalizing problems and prosocial behaviors than parental ratings at similar ages.“
